# “Dose of the day” based on cone beam computed tomography and deformable image registration for lung cancer radiotherapy

**DOI:** 10.1002/acm2.12793

**Published:** 2019-12-09

**Authors:** Zilong Yuan, Yi Rong, Stanley H. Benedict, Megan E. Daly, Jianfeng Qiu, Tokihiro Yamamoto

**Affiliations:** ^1^ Department of Radiation Oncology University of California Davis Comprehensive Cancer Center Sacramento CA USA; ^2^ Department of Radiology Hubei Cancer Hospital Tongji Medical College Huazhong University of Science and Technology Wuhan China; ^3^ Medical Engineering and Technology Research Center Shandong First Medical University & Shandong Academy of Medical Sciences Taian China

**Keywords:** adaptive radiotherapy, CBCT, dose of the day, virtual CT

## Abstract

**Purpose:**

Adaptive radiotherapy (ART) has potential to reduce toxicity and facilitate safe dose escalation. Dose calculations with the planning CT deformed to cone beam CT (CBCT) have shown promise for estimating the “dose of the day”. The purpose of this study is to investigate the “dose of the day” calculation accuracy based on CBCT and deformable image registration (DIR) for lung cancer radiotherapy.

**Methods:**

A total of 12 lung cancer patients were identified, for which daily CBCT imaging was performed for treatment positioning. A re‐planning CT (rCT) was acquired after 20 Gy for all patients. A virtual CT (vCT) was created by deforming initial planning CT (pCT) to the simulated CBCT that was generated from deforming CBCT to rCT acquired on the same day. Treatment beams from the initial plan were copied to the vCT and rCT for dose calculation. Dosimetric agreement between vCT‐based and rCT‐based accumulated doses was evaluated using the Bland‐Altman analysis.

**Results:**

Mean differences in dose‐volume metrics between vCT and rCT were smaller than 1.5%, and most discrepancies fell within the range of ± 5% for the target volume, lung, esophagus, and heart. For spinal cord D_max_, a large mean difference of −5.55% was observed, which was largely attributed to very limited CBCT image quality (*e.g.*, truncation artifacts).

**Conclusion:**

This study demonstrated a reasonable agreement in dose‐volume metrics between dose accumulation based on vCT and rCT, with the exception for cases with poor CBCT image quality. These findings suggest potential utility of vCT for providing a reasonable estimate of the “dose of the day”, and thus facilitating the process of ART for lung cancer.

## 1 INTRODUCTION

Radiotherapy is a widely used treatment option for unresectable or inoperable non‐small cell lung cancer (NSCLC) patients. Although significant progress has been made in radiotherapy for lung cancer in recent decades, improving clinical outcomes for NSCLC is still challenging.^1^ Dose escalation is one of the potential strategies to improve outcomes, but it may increase normal tissues toxicities,^2^, ^3^ for example, pneumonitis, pulmonary fibrosis, and cardiac injury, among others. A phase III randomized trial failed to demonstrate a survival benefit to dose escalation,^1^ with speculation that normal tissue toxicity may have increased deaths with high‐dose RT.^4^ It has been proposed that as tumor shrinks during the course of treatment, adaptive radiotherapy (ART) may be beneficial in reducing normal tissue toxicities and may allow safe dose escalation.^5^

While ART is often done with repeat planning CT (rCT), utilizing cone beam computed tomography (CBCT) for estimating the “dose of the day” has been an attractive research topic since they are readily available along the treatment course. However, direct use of CBCT in dose calculation is limited by inferior image quality and thus inaccurate Hounsfield units (HU).^6^, ^7^ Methods to mitigate or resolve this issue include image correction^8^, ^9^, ^10^, ^11^ and image deformation.^12^, ^13^, ^14^, ^15^, ^16^, ^17^, ^18^, ^19^, ^20^, ^21^ The former has shown promises, yet dose calculation inaccuracy still varies by up to 5% in phantom 7and patient studies of various sites.^22^, ^23^, ^24^ The latter approach, which creates deformed CT images from the initial planning CT (pCT) to CBCT, has potential to maintain anatomical information from CBCT while mapping accurate HU information from the pCT. Previous studies have shown promising results for estimating the “dose of the day” based on deformed CT images.^15^, ^17^, ^18^, ^19^, ^25^ Marchant et al.^15^ found that less than 0.5% mean dose errors can be achieved with the deformed CT images for lung cancer patients, when compared with pCT. Veiga et al.^18^ also demonstrated that the dose difference between deformed CT and rCT images for head and neck patients treated with intensity modulated radiotherapy (IMRT) was generally less than 2%. Same group also reported 3.4 ± 2.7 mm and 12 ± 12% average difference between deformed CT and rCT for lung cancer patients receiving passive scattering proton therapy.^17^ Cole et al.^21^ also found that the dose distribution based on deformed CT matched closely the rCT dose distribution in lung cancer patients receiving conformal external beam radiotherapy. An open source deformation algorithm was used in this study, which may not be practical for clinical adoption.

Adapting from the literature,^18^, ^19^, ^21^ our present study aimed to explore dosimetric accuracy of the “dose of the day” calculated on virtual CTs for lung patients receiving nine‐field IMRT treatment using commercially available deformation image registration (DIR) algorithm from a treatment planning system. A virtual CT (vCT) was created by deforming the initial planning CT (pCT) to the simulated CBCT that was generated from deforming CBCT to the repeat planning CT (rCT) acquired on the same day. And the accuracy of the “dose of the day” calculation based on vCT images was evaluated by comparing the accumulated dose based on vCT to that of rCT acquired on the same day using Bland‐Altman analysis with dosimetric parameters.

## 2 MATERIALS AND METHODS

### 2.1 Patients and imaging

This study investigated twelve patients with stage III NSCLC receiving nine‐field IMRT with a prescription dose of 60 Gy delivered in 30 fractions. Each patient underwent an rCT scan after 10 fractions as part of an ongoing clinical trial approved by the University of California Davis institutional review board (NCT02308709). Daily CBCT images were also performed for patient positioning and target localization. All pCT and rCT images were acquired during patient’s free‐breathing on a Philips 16 slice Brilliance Big Bore multi‐slice CT (Philips Medical Systems, Eindhoven, The Netherlands) with 120 kVp, 120 mA, and 2 mm slice thickness. Maximum intensity projection (MIP) CT was created based on the 10‐phase 4D CT for ITV delineation. The CBCT images were acquired on the kV x‐ray imaging system on the two matching Elekta Synergy® linear accelerator systems (Elekta AB, Stockholm, Sweden). CBCT scanning parameters include 120 kV, 40 mA tuber potential, 40 ms exposure time, 2.5 mm reconstructed slice thickness with 410 × 410 mm field of view (FOV), except one case with 20 mA and 20 ms. Note that only the CBCT images acquired on the same day with the rCT were used in this study.

### 2.2 Virtual CT with deformable image registration

**Figure 1:**
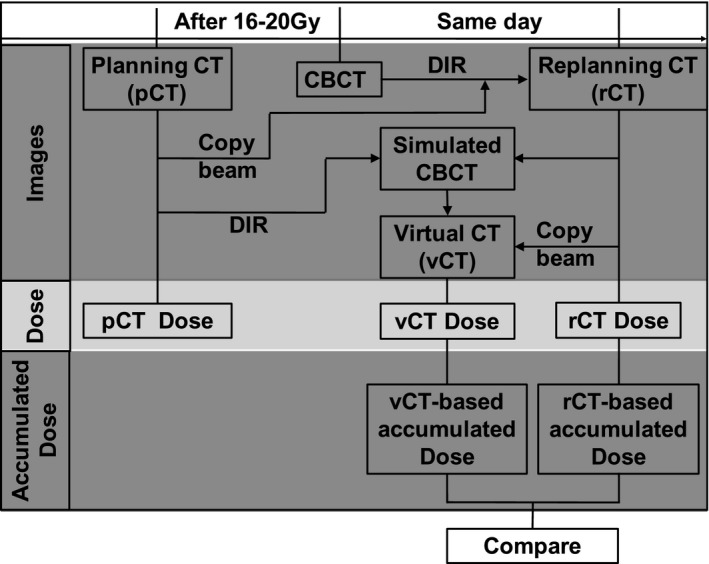
The approach of “dose of the day” calculation based on CBCT and DIR. CBCT, cone beam CT; DIR, deformable image registration.

The Demons DIR algorithm of the Pinnacle^3^ treatment planning system (research version 9.7, Philips Radiation Oncology Systems, Fitchburg, WI) was used in this study. This algorithm performs image deformation through matching image intensity with the assumption that pixels representing the same anatomical point on each image have the same image intensity values.^26^ The rCT and its corresponding CBCT were acquired on the same day, therefore, should have similar external contour and internal anatomy. Nevertheless, they may still present small organ deformation and volume variation, due to respiratory motion, positioning deviations, etc. In order to minimize such differences, a simulated CBCT image was first created by deforming CBCT to rCT (The workflow is shown in Fig. 1). Then the pCT was deformed to the simulated CBCT to create vCT images.

### 2.3 Treatment planning

The initial treatment plan based on pCT was created on the Pinnacle treatment planning system for each patient in this study. The volumes of interest were manually segmented on the pCT image sets for all cases, including gross tumor volume (GTV), internal target volume (ITV), clinical target volume (CTV) and planning target volume (PTV). GTV included the gross tumor and involved nodes as defined on the planning CT images; ITV was defined as the envelope that encompassed the GTV plus the full range of tumor motion identified on the 4D CT. The CTV was generated by adding an addition 5 mm margin, shaved off anatomic barriers to tumor spread, to the ITV. The PTV was obtained by adding 5 mm margin to CTV in all directions. Organs at risk (OAR), including bilateral lungs, spinal cord, esophagus, and heart were also delineated.

### 2.4 “Dose of the day” calculations based on rCT and vCT

The initial treatment plan was performed with coplanar or non‐coplanar 6 MV photon beams on the pCT image sets. The treatment beams from the initial plan were copied and applied to the isocenters of the rCT and vCT for subsequent dosimetric study (denoted as rCT dose and vCT dose, respectively) using the collapsed cone algorithm in Pinnacle with a 3 × 3 × 3 mm^3^ dose grid (Fig. 1). Note that all beam parameters including isocenter, control points, and monitor units were kept identical to the initial treatment plan in this dose recalculation step.

### 2.5 Dose accumulation

The vCT‐based accumulated dose was also performed with the Demons DIR algorithm to warp the vCT dose distributions as well as vCT to pCT image sets. It was presumed that the pCT plan was delivered in the first 10 fractions, followed by delivery of the vCT plan or rCT for the rest 20 fractions, therefore, accumulated dose was achieved by summing 10 times of pCT dose with 20 times of vCT or rCT dose. And rCT‐based accumulated dose performed by the same approach was used as reference to access the accuracy of vCT‐based accumulated dose. A set of DVH metrics was evaluated in this study, including PTV‐D95% (minimum dose delivered to 95% of the PTV), lung‐V20Gy (volume receiving at least 20Gy to the lung), heart‐V45Gy (volume receiving at least 45Gy to the heart), the maximum dose (D_max_) to the spinal cord, as well as the mean doses (D_mean_) to PTV, lung‐CTV and heart.

### 2.6 Statistical analysis

Bland‐Altman analysis was used for analyzing dosimetric difference between vCT and rCT based accumulated dose for all DVH metrics. The mean differences ± 1.96 times the standard deviation (SD) were defined as the limit of agreement (LOA). Statistical analyses were performed using GraphPad Prism software (v5.0, GraphPad Software, LaJoIIA, USA).

## 3 RESULTS

### 3.1 Example of the poor and good results

**Figure 2:**
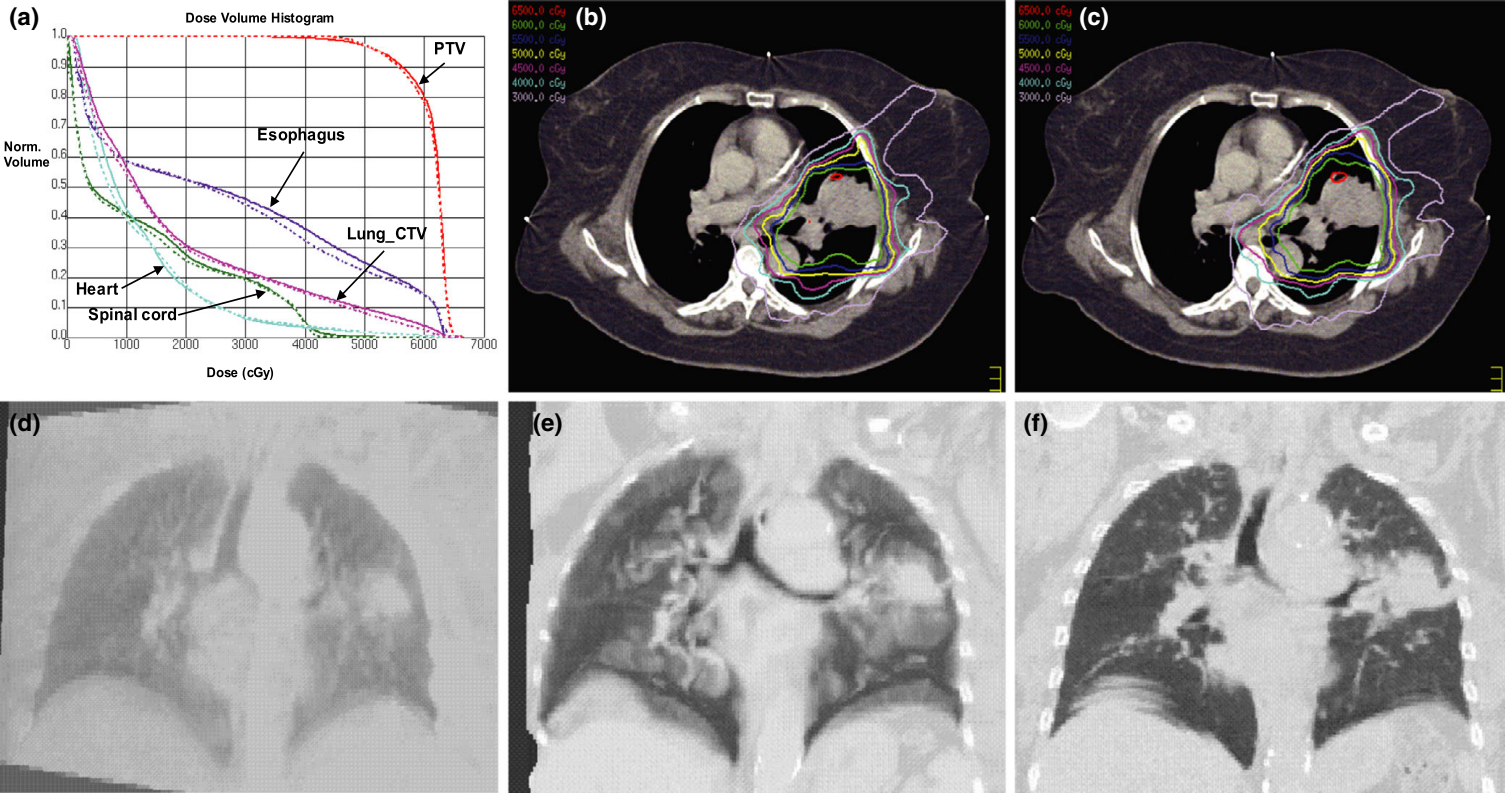
A case exhibiting poor agreement between vCT (dash line) and rCT (solid line) based accumulated doses (a), and isodose distribution comparison between vCT‐based accumulated dose (b) and rCT‐based accumulated dose (c), with their corresponding CBCT (d), vCT (e), and rCT (f).CBCT, cone beam CT.

Figure 2 shows a case exhibiting poor agreement in dosimetric comparison and their corresponding images. There are dose discrepancies between the vCT (dash line) and rCT (solid line) based accumulated doses [Fig. 2(a)] for OAR including spinal cord, lung‐CTV and esophagus. And the largest discrepancy was observed in spinal cord in particular. In addition, isodose distribution comparisons between vCT‐based [Fig. 2(b)] and rCT‐based accumulated dose [Fig. 2(c)] are poorly correlated, particularly in the spinal cord region. For this case, the CBCT of this patient has significant image quality degradation and truncation due to large patient size [Fig. 2(d)], which resulted in significant image distortion in vCT [Fig. 2(e)] and rCT images [Fig. 2(f)].

**Figure 3:**
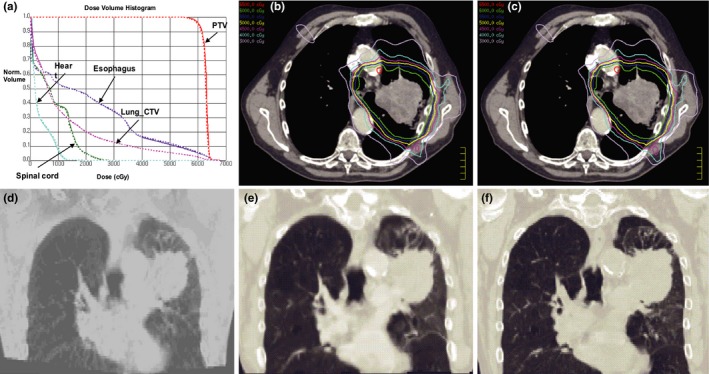
Another case exhibiting good agreement between vCT (dash line) and rCT (solid line) based accumulated doses (a), and isodose distribution comparison between vCT‐based accumulated dose (b) and rCT‐based accumulated dose (c), with their corresponding CBCT (d), vCT (e), and rCT (f). CBCT, cone beam CT.

In contrast, [Fig. 3(a)] shows a different case with good agreement between vCT (dash line) and rCT (solid line) based accumulated doses for the target volumes and OARs. The overall isodose distribution between vCT‐based [Fig. 3(b)] and rCT‐based accumulated doses [Fig. 3(c)] were comparable. Superior CBCT image quality ([Fig. 3(d)]) led to small differences between vCT [Fig. 3(e)] and rCT [Fig. 3(f)].

### 3.2 The comparison of accumulated dose for target and OAR

**Figure 4:**
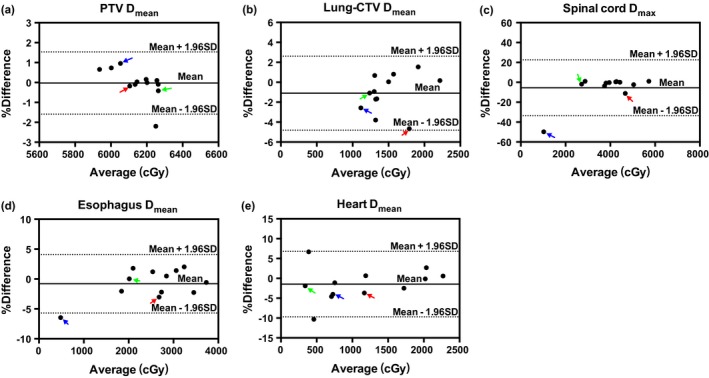
Bland‐Altman plots of percentage difference of the two accumulated dose (y‐axis) against mean accumulated dose (x‐axis), with mean percentage difference (bias) (solid line) and the limits of agreement) (dash line) for PTV D_mean_ (a), Lung‐CTV D_mean_ (b), Spinal cord D_max_ (c), Esophagus D_mean_ (d), and Heart D_mean_ (e). CTV, clinical target volume; PTV, planning target volume.

Figure 4 and Table 1 show the Bland‐Altman plots and the LOA of percentage difference between two accumulated doses based on vCT and rCT. Considering the redundancy of the data, only a part of DVH metrics including PTV (D_mean_), Lung‐CTV (D_mean_), spinal cord (D_max_), esophagus (D_mean_), and heart (D_mean_), in evaluating lung plans are shown in Fig. 4. The mean dose differences over 12 patients are smaller than 1.5% for all evaluated metrics, except for the spinal cord D_max_. As shown in Panel A, the mean difference for PTV D_mean_ is −0.03%, with LOA between −1.59% and 1.53%. One outlier is observed at −2.20%. The mean difference of Lung‐CTV D_mean_ is −1.10%, with LOA at (−4.81%, 2.61%), as shown in Panel B. Spinal cord D_max_ exhibits a high mean difference of −5.55%, with LOA at (−33.61%, 22.51%), as shown in Panel C, two subjects with −49.74% (blue arrow) and −11.13% (red arrow) differences can also be observed; discarding these high difference subjects, acceptable mean differences at −0.57% and LOA at (−3.74%, 2.59%) for spinal cord D_max_ can be achieved. Panel D and E also show −0.8% and −1.46% mean difference for esophagus D_mean_ and heart D_mean_, with a LOA at (−5.70%, 4.10%) and (−9.72%, 6.79%), respectively. Results also show other outliers for esophagus D_mean_ and heart D_mean_, with −6.46% and −10.30% differences, respectively, but with a good agreement in comparison of absolute differences (not shown in the paper).

**Table 1:** The mean percentage differences and limits of agreement of accumulated dosimetric parameters based on vCT and rCT.

	Mean percentage difference	SD	Limits of Agreement
PTV D_mean_	−0.03%	0.80%	−1.59% to 1.53%
Lung‐CTV D_mean_	−1.10%	1.89%	−4.81% to 2.61%
Spinal cord D_max_	−5.55%	14.32%	−33.61% to 22.51%
Esophagus D_mean_	−0.80%	2.50%	−5.70% to 4.10%
Heart D_mean_	−1.46%	4.21%	−9.72% to 6.79%

Abbreviations: CTV, clinical target volume; PTV, planning target volume; SD, standard deviation.

It should be noted that the poor case shown in Fig. 2 also corresponds to the red arrows in Fig. 4, which show large differences with −11.13% [Fig. 4(c)] and −4.67% [Fig. 4(b)] in spinal cord D_max_ and lung‐CTV D_mean_, respectively. Dose difference in esophagus D_mean_ and heart D_mean_ are −3.03% and −3.69%, respectively (as pointed out by red arrows in [Fig. 4(d)‐(e)]. While the good case shown in Fig. 3 which indicated by the green arrows in Fig. 4, only show less than 2% difference for all dosimetric parameters.

## 4 DISCUSSION

The vCT image sets created in this study allows for accurate accumulated dose calculation, comparable to the accumulated dose based on the rCT. Results show less than 1.5% mean differences for most dose‐volume metrics between the vCT and rCT based accumulated doses. Mean dose difference (−5.55%) of spinal cord Dmax was observed in two cases with poor CBCT image quality, which subsequently cause large DIR errors between CT and CBCT images. The approach of using the “dose of the day” calculation in this study was partly based on the studies reported by Veiga et al.^18^, ^19^ and Cole et al.,^21^ which show less than 2% dose difference for head and neck patients,^18^, ^19^ and close match for lung cancer patients^21^ when comparing vCT with rCT doses. Furthermore, most studies on accumulated dose were done evaluating DIR based accumulated dose from rCT (warping dose from rCT to pCT）vs. initial plan dose.^27^, ^28^ For example, Andersen et al.^27^ found that deviations between DIR‐based dose accumulations from rCT and the initial plan dose for prostate were substantial (Range: −0.5 to 2.3 Gy for *D*_2%_ and −9.4 to 13.5 Gy for *D*_mean_), whereas deviations between DIR‐based accumulations and DVH summation were small and well within 1 Gy. Tsudou et al.^28^ reported that the dose to parotids for dose accumulation from rCT was higher than the initial plan by 8.0% and 6.8% for ipsilateral and contralateral parotids in head and neck patients. Veiga et al.^25^ also investigated accumulated dose uncertainties using deformed images from pCT to CBCT for head and neck patients. However, none have investigated the accuracy of accumulated dose calculation based on vCT compared to that of rCT. Results shown in this study demonstrated vCT has the potential utility to provide a reasonable estimate for “dose of the day” calculation.

The Pinnacle’s Demons algorithm used in this study has been validated by other groups for CT‐CBCT image deformation.^29^, ^30^, ^31^ Visual inspection was used for validating DIRs in this study. We found from this study that the critical limiting factor to overall dose accumulation accuracy is CBCT image quality, which would subsequently affect DIR results’ accuracy. As shown in the first example, image truncation was observed in CBCT image [Fig. 2(d)] on the right side of the patient body, thus large differences between vCT vs. rCT [Figs. 2(e) and Fig. 2(f)] images. Poor CBCT image quality caused by high scatter contamination due to large patient size and projection truncation may propagate to less ideal DIR results. These DIR errors may subsequently introduce high dose deviation in spinal cord D_max_ and lung‐CTV D_mean_, as shown in [Fig. 4(c) and (b)] (red arrows). In addition, it has been observed that vCT often associated with greater truncation in the right chest wall region compared to their corresponding original CBCT images, which may be attributed to a chain of error propagation during the deformation process. High image noise and low image contrast may be introduced to the simulated CBCT images in the same region when deforming CBCT to rCT, followed by mis‐registration errors introduced to the process of deforming pCT to simulated CBCT, which therefore may lead to higher truncation artifacts in vCT images. Note that image truncation on vCT did not affect dose calculation since there were no beams passing through the truncated regions. Another case with highest difference for spinal cord D_max_ (−49.74%, Fig. 4(c), blue arrow) is also associated with poor CBCT image quality. Similar image artifacts are observed in this case due to large patient size, truncation, and inappropriate choices of imaging parameters (20mA tuber potential and 20ms expose time).

Previous studies have shown that truncated artifacts, patient size, and imaging parameters can affect CBCT image quality.^32^ Zhen et al. pointed out that the missing information with truncated CBCT images introduced incorrect deformation when a conventional DIR algorithm is utilized, especially for intensity based algorithms.^33^ Meanwhile, low imaging parameters with large patient size may result in more scatters. Wood et al.^34^ demonstrated that CBCT image signal‐to‐noise ratio drops with increased phantom sizes. Veiga et al.^19^ also pointed out large body size for CBCT imaging may be the main source of error in recalculation proton dose on CBCT. In contrast, small difference between vCT [Fig. 3(e) and rCT Fig. 3(f)] can be observed for superior CBCT image (Fig. 3(D)). Dose difference of less than 2% can be observed for all evaluated metrics (Fig. 4, green arrows), DVH and isodose distribution comparison [Fig. 3(a)‐(c)]. In such a retrospective setting, we are limited to previously acquired images. We aim to improve our clinical protocol in terms of CBCT scan setting in our future prospective studies.

Despite the promising results of the present study, there are inherent limitations. First, due to the limited scanning range and truncation in the CBCT, lung D_mean_ between vCT and rCT dose was not evaluated without the whole lung contour. Second, considering the slow gantry motion while acquiring CBCT image, to the study may be improved if rCT was created as AveCT from 4D CT for the comparison with CBCT. Third, statistical analysis cannot be achieved in this study due to limited number of patients included. A follow‐up study with large patient sample and standardized imaging parameters is warranted to further identify uncertainties in using CBCT and DIR for “dose of the day” calculation.

## 5 CONCLUSION

The accuracy of “dose of the day” calculation based on vCT was evaluated by comparing vCT with rCT based accumulated dosimetric parameters. The vCT created in this study can be used to reasonably estimate the “dose of the day” calculation for lung cancer patients with mean difference smaller than 1.5% for most accumulated dose‐volume metrics. The “dose of the day” also has the potential to become a very useful tool for ART. Critical to this calculation approach is CBCT image quality, which was found to be the main contributing factor to less ideal vCT creation, and thus less accurate dose accumulation.

## CONFLICTS OF INTEREST

The authors declare no conflicts of interest.
